# Predictors of Poor Pregnancy Outcomes Among Antenatal Care Attendees in Primary Health Care Facilities in Cross River State, Nigeria: A Multilevel Model

**DOI:** 10.1007/s10995-016-1965-5

**Published:** 2016-03-23

**Authors:** Soter Ameh, Omokhoa A. Adeleye, Caroline W. Kabiru, Thomas Agan, Roseline Duke, Nkese Mkpanam, Doris Nwoha

**Affiliations:** Department of Community Medicine, College of Medical Sciences, University of Calabar, Calabar, Cross River State Nigeria; Medical Research Council/Wits Rural Public Health and Health Transitions Research Unit (Agincourt), School of Public Health, Faculty of Health Sciences, University of the Witwatersrand, Johannesburg, South Africa; Department of Community Health, College of Medical Sciences, University of Benin, Benin City, Edo State Nigeria; African Population and Health Research Center, Nairobi, Kenya; Department of Obstetrics and Gynaecology, College of Medical Sciences, University of Calabar, Calabar, Cross River State Nigeria; Department of Ophthalmology, College of Medical Sciences, University of Calabar, Calabar, Cross River State Nigeria; Department of Community Medicine, University of Calabar Teaching Hospital, Calabar, Cross River State Nigeria

**Keywords:** “Risk Approach” strategy, Antenatal care (ANC), Primary health care (PHC), Urban–rural, Cross River State, Nigeria

## Abstract

*Objectives* Pregnancy carries a high risk for millions of women and varies by urban–rural location in Nigeria, a country with the second highest maternal deaths in the world. Addressing multilevel predictors of poor pregnancy outcomes among antenatal care (ANC) attendees in primary health care (PHC) facilities could reduce the high maternal mortality rate in Nigeria. This study utilised the “Risk Approach” strategy to (1) compare the risks of poor pregnancy outcomes among ANC attendees by urban–rural location; and (2) determine predictors of poor pregnancy outcomes among ANC attendees in urban–rural PHC facilities in Cross River State, Nigeria. *Methods* A cross-sectional survey was conducted in 2011 among 400 ANC attendees aged 15–49 years recruited through multistage sampling. Data on risk factors of poor pregnancy outcomes were collected using interviewer-administered questionnaires and clinic records. Respondents were categorised into low, medium or high risk of poor pregnancy outcomes, based on their overall risk scores. Predictors of poor pregnancy outcomes were determined by multilevel ordinal logistic regression. *Results* A greater proportion of the women in the rural areas were below the middle socio-economic quintile (75 vs. 4 %, *p* < 0.001), had no education (12 vs. 2 %, *p* < 0.001), and were in the 15–24 age group (58 vs. 35 %, *p* < 0.001) whereas women in the urban areas were older than 35 years (10 vs. 5 %, *p* < 0.001). The women attending antenatal care in the urban PHC facilities had a low overall risk of poor pregnancy outcomes than those in the rural facilities (64 vs. 50 %, *p* = 0.034). Pregnant women in the urban areas had decreased odds of being at high risk of poor pregnancy outcomes versus the combined medium and low risks compared with those in the rural areas (OR 0.55, 95 % CI 0.09–0.65). *Conclusions for Practice* Pregnant women attending antenatal care in rural PHC facilities are more at risk of poor pregnancy outcomes than those receiving care in the urban facilities. Health programmes that promote safe pregnancy should target pregnant women in rural settings.

## Significance

*What is already known about this topic*? One-level socio-demographic and obstetrical factors that predispose women to poor pregnancy outcomes have been reported in Nigeria, a country with the second highest maternal mortality in the world, and with varying levels of maternal mortality across its geopolitical regions. However, there is paucity of a multilevel data to show how a combination of socio-demographic, obstetrical and facility factors predict poor pregnancy outcomes in Nigeria.

*What this study contributes to this topic?* This study used multilevel modelling to measure individual, facility, and area risk factors of poor pregnancy outcomes for the purpose of generating evidence-based and locally-relevant strategies that could have clinical application for improving maternal and child health care in the study setting. The socio-demographic features that characterised rural-dwelling women compared to urban dwellers also characterised the risks of poor pregnancy outcomes in this study. Respondents receiving ANC in urban PHC facilities were less likely to be at risk of poor pregnancy outcomes hence the need to target women attending ANC in rural settings where the risks of poor pregnancy outcomes is higher.

## Introduction

About 800 women die daily from pregnancy-or childbirth-related complications around the world. In 2013, there were an estimated 289,000 maternal deaths, a decline of 45 % from the 1990 estimate [31]. Approximately 99 % of these deaths occurred in low- and middle-income countries (LMICs), with sub-Saharan Africa (SSA) alone accounting for 62 % (179,000) of the deaths [31]. Although the global maternal mortality ratio declined by 2.6 % per year, this is far from the annual decline of 5.5 % required to achieve the fifth Millennium Development Goal (MDG) of improving maternal health [31]. Only seven LMICs are expected to achieve the MDG 5 target of a 75 % reduction in the maternal mortality ratio by 2015 [14]. Two countries accounted for one-third of all global maternal deaths in 2013: India at 17 % (50,000) and Nigeria at 14 % (40,000) [31], suggesting that pregnant women in these LMICs have high risk pregnancies.

“Around the world, societies expect women to bear children, and honour them for their roles as mothers. Yet in most of the world, pregnancy and childbirth are a perilous journey” [22]. This perilous journey is worsened by inequities in health, which is a reflection of social injustice that varies by gender, social class, race, and urban–rural location [22]. Each pregnancy and childbirth is a risky and potentially fatal experience for hundreds of millions of women worldwide outside of a small number of privileged countries that have succeeded in reducing maternal mortality to close to zero (Gendercide Watch [9]. There are disparities in the lifetime risk of death varying between high income countries and LMICs, with the probability of a 15 year old woman dying from a maternal cause being 1 in 3700 in the former compared with 1 in 160 in the latter [30].

Within LMICs, there is a disproportionately higher risk of maternal morbidity and mortality for women living in rural areas than for those living in urban areas [30]. The high risk of maternal morbidity and mortality in rural settings may stem from a higher total fertility rate (TFR)—the average number of children a woman would bear by the end of her childbearing years. In Nigeria, the TFR is higher in rural areas (6.2) than in urban areas (4.7) [18], suggesting that women living in rural areas have higher numbers of pregnancies and are therefore more prone to risks associated with poor pregnancy outcomes than their urban counterparts. A study of high-risk pregnancies in urban and rural communities in central Ethiopia revealed that the women in the rural areas had more pregnancy-related risks than those in the urban areas [10].

Total fertility rate ranges from 4.3 to 6.7 across the north-east, north-west, north-central, south-east, south-west and south–south geo-political regions of Nigeria. Of the six states in the south–south region, Cross River State has the highest percentage of teenagers with an unmet need for family planning (30.8 %) and who have begun childbearing (18.4 %); and, the third highest percentage of teenagers pregnant with their first child (1.4 %; [18].

It is reported that urban–rural disparities exist in the healthcare system as rural people, including pregnant women, have limited access to skilled health personnel (Gendercide Watch [9, 30]. The urban–rural differences in maternal mortality among Nigerian women may also stem from the unequal distribution of quality healthcare services and infrastructure, which favours urban areas [2]. For example, the 2013 National Health and Demographic Survey (NHDS) showed that Nigerian urban-dwelling women were more likely to receive care from a skilled health provider than their rural counterparts (86 vs. 47 %, respectively; [18].

The impact of pregnancy-related complications on infants and children cannot be overstated as 4.3 million infants die during the first month of life and a million or more children are left motherless [22]. Maternal orphans are less likely to receive adequate healthcare or to access education and are up to 10 times more likely to die within 2 years than children with two living parents [22].

Pregnancy-related complications can be prevented by early identification of factors that put a pregnancy at risk [12]. The World Health Organization (WHO) developed the “Risk Approach” strategy as a managerial tool to improve maternal and child healthcare through rational distribution of existing resources [29]. In this strategy, a scoring system is used to identify individuals at the greatest risk of poor pregnancy outcomes [29]. The seven factors outlined in the “Risk Approach” strategy are poor medical and/or obstetric history; high parity (delivery of five or more infants who achieved a gestational age of ≥28 weeks); very young or older maternal age (below 18 or above 35 years); short birth interval (<2 years between the last pregnancy and index pregnancy); low income; single marital status; and low educational level. A poor medical history includes any of the following: hypertension, insulin-dependent diabetes, renal disease, and cardiac disease, while a poor obstetric history includes a history of stillbirths, spontaneous abortions, and hospital admission for pregnancy-induced hypertension or pre-eclampsia/eclampsia (11). Although the majority of these risk factors are neither direct nor indirect causes of maternal mortality, they are risk factors for poor pregnancy outcomes.

There is literature evidence to support the use of the WHO’s “Risk Approach” strategy as a valid and a reliable tool for assessing risky pregnancies in order to improve the coverage of quality of maternal and child/family planning services based on the measurement of individual and community risks. This tool is still being used for reducing pregnancy-related morbidity and mortality many years after the strategy was introduced [10, 24, 33]. In Malaysia, the risk approach involves the use of four colour codes denoting a range of severity of obstetric problems and providing a practical guide to nursing staff which enables them to identify cases for referral to a physician. The nursing staff use a checklist to facilitate the early detection of risks and complications in the antenatal period [24].

The latest Antenatal Care (ANC) policy in Nigeria follows the WHO’s Focused Antenatal Care (FANC) strategy. This strategy emphasises quality individualised care during each visit instead of focussing on the number of visits emphasised in traditional ANC. The FANC strategy recommends at least four ANC visits for women without risks of complications, while those with conditions beyond the scope of basic care may require additional visits or referrals [18, 25]. Despite the introduction of the FANC strategy, the traditional ANC approach which uses the “Risk Approach” strategy for the early detection of women at risk of poor pregnancy outcomes [29] is still commonly used for the monitoring of pregnancies because the FANC strategy is yet to be implemented in many PHC facilities in Nigeria.

One-level socio-demographic and obstetrical factors that predispose women to poor pregnancy outcomes have been reported in Nigeria, a country with the second highest maternal mortality in the world [31] and with varying levels of maternal mortality across its geopolitical regions [18]. Late ANC booking [1, 7, 8, 11], low education and high parity [8] are risk factors for poor pregnancy outcomes in Nigeria. However, there is paucity of a multilevel data to show how a combination of socio-demographic, obstetrical and facility factors predict poor pregnancy outcomes in Nigeria. This is in view of the evidence that multilevel modelling is a more statistically robust approach than single-level modelling for testing associations, specifically in a clustered data to which multilevel factors are a component, as was the case in our study. Furthermore, little is known about the risk profile of ANC attendees in PHC facilities in Cross River State which has some of the worst obstetric history for teenagers in the south–south geopolitical region of Nigeria [18]. This study utilised the “Risk Approach” strategy to (1) compare the risks of poor pregnancy outcomes among ANC attendees by urban–rural location; and (2) determine the predictors of poor pregnancy outcomes among ANC attendees in PHC facilities in an urban and a rural Local Government Areas (LGAs) in Cross River State.

## Methodology

### Study Design and Setting

This was a cross-sectional survey that was conducted between September and December 2011 among women aged 15 to 49 years attending ANC in PHC facilities situated in an urban and a rural LGA of Cross River State, south–south region of Nigeria. The total population of the state in the 2006 national population and housing census was 2,892,988: males—50.9 % (1,471,967) and females—49.1 % (1,421,021) [17]. There are 18 LGAs in the state and these are unevenly distributed in the northern (5), central (6) and southern (7) senatorial or political districts. At the time this study was conducted, there were 153 PHC facilities in the seven LGAs that made up the southern senatorial district which was the study setting: Akamkpa—32, Akpabuyo—30, Bakassi—13, Calabar municipality—23, Calabar South—21, and Odukpani—34. Of these seven LGAs in the southern senatorial district, Calabar south and Calabar municipality are categorised as urban LGAs according to the urban–rural classification of LGAs in Cross River State.

### Sample Size Estimation

We calculated a minimum sample size of 200 participants for each LGA after adjusting for 10 % non-response, using a two proportion sample size formula [15] with two-sided distribution, 95 % confidence level, 90 % power, and assuming 12 % significant difference in risk status between rural (20 %) and urban (8 %) LGAs.

### Sampling Technique

Multistage sampling was used to recruit 400 pregnant women attending ANC services in PHC facilities in urban and rural LGAs. First, simple random sampling was used to select the southern senatorial district from the three senatorial districts (southern, central and northen) in the state. Second, an urban (Calabar South) and a rural (Odukpani) LGA were selected by simple random sampling from the seven (two urban and five rural) LGAs that constitute the southern senatorial district using the existing list of urban and rural LGAs in the State’s urban renewal programme. Third, 15 (six urban and nine rural) of the 55 PHC facilities in the two LGAs were randomly selected. All the 55 PHC facilities in the selected LGAs offered ANC services at the time this study was conducted. Fourth, pregnant women who met the inclusion criteria for the study were recruited by systematic sampling, using facility-specific sampling interval. Inclusion criteria were permanent residency status in the community served by the PHC facilities and having been booked for ANC irrespective of the gestational age.

### Training and Quality Assurance

Fifteen field workers and five supervisors participated in a three-day training workshop on field procedures and questionnaire administration. The questionnaire was pretested in a PHC facility that was not on the list of study sites in the main study, after which a new training session was held to review challenges. Each PHC facility was then assigned a fieldworker for data collection and a supervisor was assigned to three PHC facilities to supervise the fieldworkers. The supervisors immediately checked the completed questionnaires for errors and inconsistencies at random.

Only a few statements in the questionnaire had to be rephrased after pretesting. Quality assurance followed a two-step system after pre-testing. First, the field workers double-checked the questionnaire after an interview with a pregnant woman and; secondly, the supervisors randomly checked the questionnaires for inconsistencies and blank questions before the interviewed pregnant woman exited the health facilities.

### Variables

Individual-level data were collected using interviewer-administered questionnaires that gathered information on the socio-demographic features, obstetric characteristics, medical history, household possessions and housing characteristics. Data on the medical history of the respondents were complemented by review of clinic records. Facility-level data were collected by facility audit and review of clinic records. A score of three points was allocated for a poor medical and/or obstetrical history, three points for high parity, two points for very young (<18 years) or old (>35 years) maternal age and one point each for short birth interval, low income, single marital status and low educational level in accordance with the scoring system in the “Risk Approach” strategy [29]. Of a total of 12 possible points, women with 0–2, 3–5 or ≥6 points were categorised into low, medium or high risk of poor pregnancy outcomes (the composite ordinal outcome variable), respectively [29].

The wealth index was used as a proxy for income in this study. This is in view of evidence that self-reported measures of income are unreliable in LMICs due to the reluctance to reveal such information along with the myriad of transactions undertaken by self-employed people; thus making it unlikely for respondents to give an accurate account of their income [5]. The wealth index of the respondents was constructed from 22 variables on household possessions and housing characteristics using the principal components analysis (PCA) technique [27]. Wealth index was categorised into quintiles in ascending order of lowest, middle low, middle, middle high, and highest socio-economic status (SES). Respondents below the middle quintile were reported to be of low SES.

### Statistical Analysis

We hypothesised that pregnant women receiving antenatal services in urban PHC facilities are less likely to be at high risk of poor pregnancy outcomes. STATA^R^ version 12 was used for statistical analysis. Statistical analysis was done at 5 % level of significance. The data set was declared as survey data by using the ‘svyset’ syntax to enable adjustment of estimates due to the complex multistage sampling design and the clustered nature of the data; hence, the use of design effect in the adjustment of estimates. The Pearson Chi squared test was used to compare respondents’ risks of poor pregnancy outcomes by urban–rural LGA. Predictors of poor pregnancy outcomes were examined at three levels: individual (e.g. socio-demographic and obstetric features), facility (e.g. resources and services) and area (urban vs. rural location of facilities) factors. Predictors of poor pregnancy outcomes were determined by ordinal logistic regression analysis. The “meologit” Stata syntax was used to fit a mixed-effect multilevel ordinal logistic regression model which had two effects: fixed effects describing the intercepts and slopes of the population as a whole and random effects showing how the intercepts and slopes vary across patients nested within health facilities and the latter nested within urban or rural areas. The cut-off point for the unadjusted regression analysis was set at 20 % significance level and the relationships between independent variables and poor pregnancy outcomes were analysed by forward selection. Variables with confidence intervals including the null value of one were excluded for analysis in the adjusted regression model. Variables that significantly (confidence intervals excluding the null value of one) predicted poor pregnancy outcomes in the unadjusted analysis were included in the multivariate adjusted model and analysis was done at 5 % significance level. Variables (age; education, SES, marital status, parity, birth interval, and medical and obstetric conditions) from which the composite outcome variable (risk of poor pregnancy outcomes) was derived were excluded as independent variables in the regression analysis because of multicolinearity of these aforementioned variables with the outcome variable.

### Ethical Clearance

Ethical clearance for this study was obtained from the Cross River State Research Ethics Committee. Permission to conduct this study was received from the nurse-in-charge of the selected PHC facilities and written informed consent was obtained from all prospective participants prior to administering the study tools. Our research was conducted in accordance with prevailing ethical principles.

## Results

Table 1 shows the respondents’ socio-demographic characteristics by area of residence. A greater proportion of the women attending ANC in PHC facilities situated in the rural LGA were below the middle socio-economic quintile (75 vs. 4 %, *p* < 0.001), had no education (12 vs. 2 %, *p* < 0.001), and were in the 15–24 age group (58 vs. 35 %, *p* < 0.001), whereas more of the women attending ANC in PHC facilities situated in the urban LGA were older than 35 years (10 vs. 5 %, *p* < 0.001).

**Table 1:** Socio-demographic characteristics of the respondents attending ANC in urban and rural PHC facilities (N = 400)

Variable	Urban LGA	Rural LGA	Total	Design effect adjusted
	n (%)	n (%)	n (%)	*p* value
Age (years)				
15–24	71 (35.5)	116 (58.0)	187 (46.8)	<0.001*
25–34	109 (54.5)	74 (37.0)	183 (45.7)	
35–49	20 (10.0)	10 (5.0)	30 (7.5)	
Education				
None	4 (2.0)	24 (12.0)	28 (7.0)	<0.001*
Primary	51 (25.5)	36 (18.0)	87 (21.7)	
Secondary	100 (50.0)	126 (63.0)	226 (56.5)	
Tertiary	45 (22.5)	14 (7.0)	59 (14.8)	
Religion				
Christianity	200 (100.0)	199 (99.5)	399 (99.7)	0.498
Islam	0 (0.0)	1 (0.5)	1 (0.3)	
Marital status				
Unmarried	47 (23.5)	32 (16.0)	79 (19.7)	0.446
Married	153 (76.5)	157 (78.5)	310 (77.5)	
Other^a^	0 (0.0)	11 (5.5)	11 (2.8)	
Ethnicity				
Efik	42 (21.0)	77 (38.5)	119 (29.8)	0.010*
Ibibio	78 (39.0)	67 (33.5)	145 (36.2)	
Anang	38 (19.0)	45 (22.5)	83 (20.8)	
Other^b^	42 (21.0)	11 (5.5)	53 (13.2)	
Occupation				
None	26 (13.0)	52 (26.0)	78 (19.5)	1.000
Farming	11 (5.5)	42 (21.0)	53 (13.2)	
Trading	85 (42.5)	73 (36.5)	158 (39.5)	
Fishing	7 (3.5)	1 (0.5)	8 (2.0)	
Civil service	48 (24.0)	18 (9.0)	66 (16.5)	
Other^c^	23 (11.5)	14 (7.0)	37 (9.3)	
Socio-economic status				
Highest	66 (33.0)	14 (7.0)	80 (20.0)	<0.001*
Middle high	69 (34.5)	11 (5.5)	80 (20.0)	
Middle	57 (28.5)	25 (12.5)	82 (20.5)	
Middle low	7 (3.5)	84 (42.0)	91 (22.7)	
Lowest	1 (0.5)	66 (33.0)	67 (16.8)	

^a^Other = divorced, separated and widowed

^b^Other = ethnic groups other than Efik, Ibiobio and Anang

^c^Other = students and artisans

* Statistically significant p-value

A significantly lower proportion of women in the rural LGA than those in the urban LGA booked for ANC in their first trimester (6 vs. 10 %, *p* < 0.001)—Table 2.

**Table 2:** Obstetric characteristics of the respondents attending ANC in urban and rural PHC facilities (N = 400)

Variable	Urban LGA	Rural LGA	Total	Design effect adjusted
	n (%)	n (%)	n (%)	*p* value
Booking for antenatal care (months)				
1–3	20 (10.0)	20 (10.0)	32 (8.0)	<0.001*
4–6	156 (78.0)	163 (81.5)	319 (79.8)	
7–9	24 (12.0)	25 (12.5)	49 (12.2)	
Gestational age at time of interview (months)				
4–6	122 (61.0)	127 (63.5)	249 (62.2)	<0.001*
7–9	78 (39.0)	73 (36.5)	151 (37.8)	
Total number of pregnancies				
1	73 (36.5)	93 (46.5)	166 (41.5)	1.000
2–4	93 (46.5)	85 (42.5)	178 (44.5)	
≥5	34 (17.0)	22 (11.0)	56 (14.0)	
Total number of miscarriages (n = 234)				
0	83 (65.3)	75 (70.1)	158 (67.5)	1.000
1	35 (27.6)	29 (27.1)	64 (27.4)	
2	9 (7.1)	3 (2.8)	12 (5.1)	

* Statistically significant *p* value

With respect to the seven risk factors assessed using the “Risk Approach” strategy (Table 3), a greater proportion of women from the rural LGA than those from the urban LGA had a maternal age <18 or >35 years (16 vs. 5 %, *p* < 0.001), had no education (12 vs. 2 %, *p* < 0.001), were of low socio-economic status (75 vs. 4 %, *p* = 0.013) and reported a birth interval <2 years between their last pregnancy and index pregnancy (47 vs. 37 %, *p* = 0.032). The total composite risk scores showed that a greater proportion of women in the rural LGA than in the urban LGA had a high or medium risk of poor pregnancy outcomes (51 vs. 36 %, *p* = 0.034).

**Table 3:** Risks of poor pregnancy outcomes among respondents attending ANC in urban and rural PHC facilities (N = 400)

Variable	Urban LGA	Rural LGA	Total	Design effect adjusted
	n (%)	n (%)	n (%)	*p* value
Poor medical or obstetrical history				
Yes	44 (22.0)	32 (16.0)	76 (19.0)	1.000
No	156 (78.0)	168 (84.0)	324 (81.0)	
High parity (>5 previous births)				
Yes	34 (17.0)	22 (11.0)	56 (14.0)	0.598
No	166 (83.0)	178 (89.0)	344 (86.0)	
Age (<18 or >35 years)				
Yes	9 (4.5)	32 (16.0)	41 (10.2)	<0.001*
No	191 (95.5)	168 (84.0)	359 (89.8)	
Low SES (below the middle quintile)				
Yes	8 (4.0)	150 (75.0)	158 (39.5)	0.013*
No	192 (96.0)	50 (25.0)	242 (60.5)	
Unmarried				
Yes	47 (23.5)	32 (16.0)	79 (19.8)	0.748
No	153 (76.5)	168 (84.0)	321 (80.2)	
No education				
Yes	4 (2.0)	24 (12.0)	28 (7.0)	0.040*
No	196 (98.0)	176 (88.0)	372 (93.0)	
Short birth interval (<2 years)				
Yes	73 (36.5)	93 (46.5)	166 (41.5)	0.032*
No	127 (63.5)	107 (53.5)	234 (58.5)	
Overall risk				
High	13 (6.5)	18 (9.0)	31 (7.7)	0.034*
Medium	59 (29.5)	83 (41.0)	142 (35.5)	
Low	128 (64.0)	99 (50.0)	227 (56.8)	

* Statistically significant *p* value

In the multivariate ordinal logistic regression analysis (Table 4), ANC attendees in the urban LGA had decreased odds of being at high risk of poor pregnancy outcomes versus the combined medium and low risks compared with ANC attendees in the rural LGA (OR 0.55, 95 % CI 0.09–0.65).

**Table 4:** Predictors of poor pregnancy outcomes among ANC attendees in PHC facilities in Cross River State (N = 400)

Variable	High risk of poor pregnancy outcomes	
	Crude OR (80 % CI)	Adjusted OR (95 % CI)
*Individual factors*		
Occupation		
None	1	
Farming	0.79 (0.10–6.53)	
Trading	0.48 (0.22–1.01)	
Fishing	0.29 (0.02–3.55)	
Civil service	0.80 (0.09–4.22)	
Other	0.86 (0.11–6.48)	
^a^Booking for antenatal care (months)		
1–3	1	1
4–6	2.57 (0.12–4.61)	1.52 (0.06–2.13)
7–9	3.49 (1.87–5.98)*	2.45 (0.43–3.40)
Total number of pregnancies		
1	1	
2–4	1.15 (0.59–2.24)	
≥5	1.29 (0.34–4.89)	
^b^Age (<18 or >35 years)		
Yes		
No		
^b^No education		
Yes		
No		
^b^Unmarried		
Yes		
No		
^b^Low SES (below the middle quintile)		
Yes		
No		
^b^High parity (>5 previous births)		
Yes		
No		
^b^Short birth interval (<2 years)		
Yes		
No		
^b^Poor medical or obstetrical history		
Yes		
No		
*Facility factor*		
Referral		
No	1	
Yes	0.23 (0.01–4.12)	
*Area factor*		
^a^Location of PHC facilities		
Rural LGA	1	1
Urban LGA	0.57 (0.05–0.78)*	0.55 (0.09–0.65)*

^a^Odds ratios were adjusted in the multivariate ordinal regression model for booking for antenatal care and location of PHC facilities

^b^The effects of age; education; SES; marital status; parity; birth interval; and medical and obstetric were not examined in the univariate regression analysis as independent variables because of multicolinearity of these variables with risk of poor pregnancy outcomes, the composite outcome variable which was derived from the aforementioned variables

* Statistically significant *p*-value

## Discussion

A greater proportion of women in the rural areas were below the middle socio-economic quintile and had no education, whereas a greater proportion of women in the urban areas were older than 35 years. The women attending antenatal care in the urban PHC facilities had a lower overall risk score for poor pregnancy outcomes than those in the rural facilities. Pregnant women in the urban areas than in the rural areas were less likely to be at high risk of poor pregnancy outcomes versus the combined medium and low risks.

The maternal mortality ratio in Nigeria declined in 2013 by 50 % from the 1990 level [31]. This is indicative of the global, regional and national interventions aimed at achieving MDG 5 [31, 32]. However, these interventions may not have been adequate in reducing the existing urban–rural disparities in the risk factors for poor pregnancy outcomes. Previous studies around predictors of poor pregnancy outcomes using the “Risk Approach” strategy have not been conducted in Cross River State; therefore, this study provides the baseline evidence of the risk profile of pregnant women utilising public PHC facilities for the purposes of planning and programming of antenatal care services in the state.

A greater proportion of women in the rural areas were of low SES. Previous studies in Nigeria [19, 20] have demonstrated a significant association between low SES and anaemia in pregnancy, a risk factor for poor pregnancy outcomes. Women of low SES have also been shown to be more likely to have adverse health and pregnancy outcomes than those from higher socio-economic backgrounds [13]. A study in Nigeria showed that empowered women are more likely to have better reproductive health outcomes, with this trend varying by empowerment dimension [4]. Therefore, efforts aimed at reducing urban–rural inequalities in maternal mortality should focus on improving rural women’s socioeconomic status, through implementation of poverty-alleviating programmes.

A greater proportion of women in the rural areas had no education in this study. This is corroborated in the 2013 NDHS which showed urban–rural differences that are more pronounced at the lowest educational level. More than half (54 %) of the women in the national survey were rural women who had no education, as compared with 16 % of urban women with no education. In the light of the evidence that the risk of maternal death decreases with more years of education [8], promotion of universal basic education targeted at women in rural communities could be key to reducing the risk factors for poor pregnancy outcomes.

The women in the rural areas had shorter birth intervals between their last pregnancy and index pregnancy. This could be an indication of lower access to or uptake of contraceptives [32]. The 2013 NDHS revealed that the use of contraceptives by women in the 15–49 age category was lower in the rural areas than in the urban areas (9 vs. 27 %), and this trend was observed across all modern methods of contraception [18]. There is evidence that the use of contraceptives can be increased through information dissemination [2], as was proven in Bangladesh [13]. According to Westoff et al., *“*television exposes viewers to aspects of modern life that compete with traditional attitudes toward the family and is associated with greater use of modern contraceptive methods, with a desire for fewer children and lower fertility” [28]. The use of media campaigns to inform the use of modern contraceptives could play a significant role in reducing the proportion of women with short birth intervals in the rural areas.

Using age <18 or >35 years, our study showed that the women attending ANC in rural PHC facilities were more at risk of poor pregnancy outcomes. A previous study in rural northwest Bangladesh showed that women younger than 18 years and older than 35 years had increased risk of obstetric complications compared with those aged 19–34 years [23]. There is also evidence that age >35 years is associated with a three- to four-fold increase in maternal mortality in Nepal [3]. Obstetric risks occurring in younger age have been attributed to the inability of adolescents below 18 years to meet increased nutritional demands of pregnancy and lactation [21]. The high prevalence of early childbearing in the rural areas in this study can be attributed to early marriage and school drop-outs [16]. Programmes that delay the timing of marriage and childbearing may therefore play a key role in reducing high risks of poor pregnancy outcomes.

A significantly fewer proportion of ANC attendees in the rural PHC facilities booked for ANC in the first trimester. Previous studies in Nigeria have also reported late presentation for ANC [1, 6, 7, 18]. Late ANC booking prevents early detection of problems during pregnancy; thereby, leading to late treatment and referrals of pregnancy complications [18]. These results suggest the need to target rural women for early ANC booking.

The women attending ANC in the urban facilities were less likely to be at risk of poor pregnancy outcomes. This corroborates literature evidence that women in rural areas have more pregnancy-related risks than those in the urban areas [10, 18], a situation that has been attributed to unequal urban–rural infrastructural distribution of healthcare services and skilled healthcare workforce in Nigeria [2, 18].

The “Risk Approach” strategy is a managerial tool for the rational distribution of existing resources, based on measurements of individual and area/community risk factors that predict poor pregnancy outcomes, and for developing local strategies and determining the appropriate content of maternal and child health care. This study showed that the use of multilevel modelling to measure these risks, including facility risk factors, could be useful for the purposes of generating evidence-based and locally-relevant strategies that may have clinical application for improving maternal and child health care in the study setting and other LMICs where rural communities have been reported to have a higher risk of poor pregnancy outcomes than urban communities. Therefore, maximum utilisation and equitable distribution of ANC services and skilled healthcare workers to rural underserved areas could potentially reduce urban–rural disparities in the risk factors for poor pregnancy outcomes. Furthermore, health education programmes that focus on reducing high risk pregnancies should target pregnant women receiving ANC services in rural communities.

We determined the predictors of poor pregnancy outcomes among women utilising the traditional ANC services which assumes that more visits mean better pregnancy outcomes. But more frequent visits do not necessarily improve pregnancy outcomes [25], and are often a source of financial constraints for the women and a burden on the healthcare system [26]. In this study, the rural LGAs had more PHC facilities than the urban LGA (34 vs. 21), but the rural-dwelling women were more at risk of poor pregnancy outcomes than their urban-dwelling counterparts. This suggests that the availability of more PHC facilities in the rural LGA than in the urban LGA did not confer an advantage on the former in terms of reducing the risk of poor pregnancy outcomes. Therefore, we recommend provision of quality ANC services that uses the traditional ANC approach and tool in the study setting and elsewhere in Nigeria until such a time there is a transitioning from the usage of the traditional ANC strategy to the FANC approach which also emphasises quality individualised care during each ANC visit.

The main strength of this study was the use of multilevel modelling to measure individual, facility and area/community risk factors of poor pregnancy outcomes as outlined in the WHO’s “Risk Approach” strategy. Unlike the single-level modelling which would have focused on one component of the study tool, the multilevel approach addressed all components from which the composite scores were derived and used to measure the overall risk of poor pregnancy outcomes. Although the latest ANC policy in Nigeria recommends the use of the focused antenatal care (FANC) strategy, we did not use the FANC tool to measure the risk of poor pregnancy outcomes because the FANC strategy had not been implemented in Cross River State at the time this study was conducted. We rather used the relatively older traditional tool which is still a valid and a reliable alternative for measuring risky pregnancies. The dearth of facility-level data limited the inclusion of human and material resources as well as ANC-related clinical services in the analysis. Our study did not assess the actual health and pregnancy outcomes for the index pregnancies because we used a cross-sectional survey. We recommend future studies follow up women until birth to provide further information on pregnancy outcomes.

## Conclusions

 Our study showed that ANC attendees in the urban PHC facilities were less likely to be at risk of poor pregnancy outcomes compared with those in the rural settings. The socio-demographic features (e.g. age <18 or >35 years, little or no education and low SES) that characterised rural-dwelling women compared to urban dwellers also characterised the risks of poor pregnancy outcomes in this study. Therefore, safe pregnancy programmes should target rural women of low SES and those with no education. Efforts to reduce adolescent childbearing through public health education are also justified.

## Appendix

See Table 5.

**Table 5:** Description of variables used to identify risk factors of poor pregnancy outcomes

Variable	Description
Socio-demographic characteristics	
Young age	Pregnant women less than 18 years of age
Old age	Pregnant women greater than 35 years of age
Marital status	Single marital status (unmarried)
Educational level	No years of completed formal education
Poor medical history	Heart disease
	Rubella in first trimester
	Breech extraction
	Prolonged pregnancy (>294 days)
	Multiple pregnancy
	Prolapsed cord
	Severe anaemia
	(Pre-) eclampsia
	Caphalopelvic disproportion
	Placenta praevia
	Abruptio placenta
	Malaria
Poor obstetric history	
High parity	Delivery of five or more infants who achieved a gestational age of ≥28 weeks
Short birth interval	Interval of <2 years between the last pregnancy and index pregnancy
^a^Wealth index (Socio-economic status)	

^a^Details of how wealth index, a proxy for income, was derived are shown in the variables sub-section
